# Industry-Led Standards, Relational Contracts and Good Faith: Are the UK and Australia Setting the Pace in (Construction) Contract Law?

**DOI:** 10.1007/s10991-022-09307-5

**Published:** 2022-07-20

**Authors:** David Christie, Séverine Saintier, Jessica Viven-Wilksch

**Affiliations:** 1 Robert Gordon University, Aberdeen, United Kingdom; 2 Exeter University, Exeter, United Kingdom; 3 The University of Adelaide, Adelaide, Australia

**Keywords:** Construction contracts, Good faith, Relational contract, Project-centric approach, Party-centric approach

## Abstract

The law of contract is changing. “Good faith” and “relational contracts” are used by parties more than ever before in commercial disputes. Yet, their definition and what it really means to act in good faith are still unsettled in the UK and Australia, reducing the (judicial and doctrinal) utility and impact of such conceptual tools. In contrast, the construction industry is trying to move forward in policy terms. Over the last 30 years, industry-led initiatives have been working to improve collaboration. In the UK and Australia, new collaborative frameworks contain express provisions asking parties to act with mutual trust and cooperation among other collaborative schemes. Examination of the judicial approach and industry initiatives demonstrates that there is – underpinning both – a *project-centric* approach (even if that is yet to be fully recognised or articulated). It is the aim of this paper to further articulate this understanding by examining at the judicial and industry positions in the UK and Australia.

## Introduction

There has been a growing move towards acceptance of good faith and relational contracts in the common law. In the UK,^1^ in 2020, the British Institute of International and Comparative Law concept note on Covid-19^2^ and commercial contracts endorsed the view expressed extra-judicially by Mr Justice Leggatt (as he then was) in his lecture to the Commercial Bar Association in 2016 that good faith obligations are important in allowing the commercial flexibility required in modern contracts in common law countries, and beyond. (Leggatt 2016). This view might appear widely shared by the English courts; exemplified by *Yam Seng v ITC Ltd*^3^ and *Sheikh Al Nehayan v Ionnis Kent*,^4^ which applied good faith as an implied term to contract law, and *Bates v Post Office Ltd (No 3)*,^5^ which discussed the notion in the context of the emerging category of relational contracts. Yet these notions, aimed at developing flexibility, remain (stubbornly) divisive^6^ and their doctrinal impact is consequently limited (Tan 2019; Collins 2016: 37). The UK is far from alone in its struggle with those notions. In Australia, ever since *Renard Construction v Minister for Public Works*^7^ parties have been toying with good faith in contract law but without landing on anything particularly significant. The struggle also exists in the USA (MacMahon 2015).

There are echoes in the experience of the construction industry where there have been efforts in both Australia and the UK, over the last few decades, to instill a more collaborative form of working, but efforts have come up somewhat short. The recent developments in case law *ought* to provide a means to drive forward these goals but as it stands, the policy has not been fully recognised and articulated. As a result, the law has yet to meet the policy goal. We will discuss how this lack of articulation makes it difficult to advance the discussion – instead creating a vicious circle of rejection.

Why do these notions cause so much controversy? Looking at case law from both jurisdictions, a similar theme is emerging: the notions are too vague^8^ to allow clear enumeration of what they mean for the parties in terms of their rights and obligations. This vagueness, in turn, fails to give parties the necessary certainty of their position to form a protective boundary for their own interests.

This present state of affairs relies on a traditional view of contract, which focusses too narrowly on the individual parties and tests the question of what is required of them by sole reference to their own contractual self-interest. In this paper, we refer to this as a *party-centric* approach to contracts. In taking this approach, the courts fail to give effect to the bigger picture of using the concepts of the relational contract and good faith as mechanisms to add in the desired flexibility and cooperation. This comes despite a more general move in the courts to give full effect to the intentions of the parties (Robertson 2019: 231). Although the courts regularly deal with ‘open-textured’ concepts and use language which is open to some ambiguity (Hogg 2017:1673; Rowan 2021; Chen-Wishart and Dixon, 2020), good faith and relational contracts seem to be particular stumbling blocks.

This *party-centric* approach, in focusing on the parties’ contractually-defined legal obligations, is too restrictive. We argue that a better solution is to formally give effect to a *project-centric* approach, which places the parties’ individual interest within the wider lens of their shared interests in achieving the agreed common purpose of the contract. That *project-centric* approach, which allows one to decouple the parties’ individual interests from the wider purpose of the contract (Robertson 2019: 234), is embedded in the case law and is slowly being articulated through the concepts of relational contracts and good faith, but has not yet been given full effect. We further argue that the policy drive within the construction industry can be characterised as being *project-centric* in its aims and that it meets both the judicial drive (most notably led by Leggatt J (as was) in *Yam Seng* and *Sheikh Al Nehayan*, but also Fraser J in *Bates*) and the most recent doctrinal drive (Tan 2019; Gounari 2021) towards further development of the concepts of relational contracts and good faith. However, the existing reliance on *party-centric* concepts in the case law means that there is currently insufficient vocabulary for that development to happen. This lacuna is not surprising, the legal vocabulary in case law and doctrine simply reflects the traditional contract law theories and is therefore embedded in the traditional view of the law. Existing legal concepts can only be described using this traditional language and so the *party-centric* view is difficult to depart from: simply put, the words/vocabulary to describe a different approach do not exist yet.

This is of course problematic since without that vocabulary, it is difficult to articulate and develop the words to reflect and explain the multifaceted nature of those concepts away from their *party-centric* focus. Without capturing the essence of what the concepts mean, the legal understanding and language applicable to these concepts remains limited … and so, the vicious circle of rejection goes. We argue that the concepts of relational contracts and good faith help to give effect to a *project-centric* approach by providing a framework to exit this circle of rejection and provide a vocabulary which can then be used to develop the law.

The article is organised as follows. First, the debate over the notions of good faith and relational contracts is placed in its context, and in the construction industry context in particular. A second part will then show that the *project-centric* approach as articulated here is clearly embedded in the courts’ understanding of good faith and draws from existing ideas in theory but that the consequences of this need to be more fully understood. Particular reference to the construction industry will be made. A third part will then suggest next steps to develop thinking in that respect and reflect on what we consider shows a *project-centric* approach to construction contracts.

## The existence and limits of “good faith” and its role in the construction industry

Both the UK and Australia have debated the place and role of good faith in contract, its definition, and its application for a long time. Since *Carter v Boehm*, 9 the notion has appeared before the English courts without their taking a definite stance on the matter. Australian courts are also ill-at-ease with it and prefer adjudicating on other matters to avoid dealing with the concept of good faith and its application in Australian contract law.^10^

Critical voices have described the concept as a ‘legal irritant’, (Teubner 1998; White 2000) a ‘strange and worrying chameleon’ (Shalev 1992: 820, Chunlin 2010: 1), something that academics ‘can get a little over-excited about’ (Coulson LJ speaking extra-judicially, 2019) or an amorphous notion that is not needed and could in fact be dangerous to the law of contract in the UK.^11^ These discussions on good faith are not limited to the UK and Australia. Other common law jurisdictions have also tried to determine the place, if any, of good faith in contract law.^12^

In spite of all this, ‘good faith’, far from vanishing, reappears time and again in the UK and Australia. Indeed, as the idea of the relational contract has moved from ‘academic theory’^13^ to case law and legal practice, it has brought further references and discussion of ‘good faith’ along with it. The notions are inextricably linked. The wider theory should help inform the emerging understanding in practice. Moreover, “good faith” emerges in different ways in different cases whether as an implied term, a more general value or as an express term of the parties’ agreement. All of these show some attempts to recognise the ideas noted above: to try and adjust the parties’ approach to contracting.

Indeed, the ‘other-regarding’ values which are inherent in relational contracts, and are given effect to by good faith obligations, are important in this respect (Gerhart 2020). They seem to capture the zeitgeist of greater cooperation and commercial flexibility noted above. In short, good faith (understood as a standard of cooperation in achieving the agreed-upon results (Corcoran 2012: 9)) and relational contracts help to articulate the values behind the *project-centric* approach. Their lack of formal recognition and the vocabulary for articulation that would bring, therefore, fuels the circle of rejection described above.

In parallel to this, formal efforts to make the construction industry less adversarial, more flexible and more cooperative have been clear in the UK since the publication of the Latham report in the early 1990s (Latham 1994). That has led to a number of initiatives, and we suggest that the recent discussion on good faith should help in understanding those further. Writ large is the use of partnering and alliancing contracts where the economic framework of the contract leads to significant alignment of the parties’ economic incentives. Writ smaller is the use of collaborative frameworks in contracts, in particular the NEC suite (Christie 2018) – including its specific and explicit “mutual trust and cooperation” provision as the first substantive clause in the contract.^14^ This suite of contracts provides for particular communication mechanisms of ‘early warnings’ of changes, which promote early working out of risks. More recent innovations include the use of enterprise contracts, which focus on parties’ conduct emerging through the project (Mosey and Jackson 2020). Other initiatives also include early involvement of contractors in the design and planning of the project (Harvey 2018). Finally, practical efforts to improve collaboration in the industry, such as the payment mechanism, coupled with speedy dispute resolution, are found in the UK^15^, and in several Australian states (summarised in Jones Day 2020), as well as other jurisdictions including Ireland, Singapore, and Malaysia. Most recently, the UK Government’s ‘Construction Playbook’, published in December 2020 (UK Government, 2020), represents a vision of how the UK Government will engage and operate its own construction contracts, and is infused with ideas of longer-term approaches to relationship management, a focus on outcomes, and the promotion of flexible and collaborative working. The emerging jurisprudence on relational contracts increasingly resonates with the policy outcomes sought here.

A policy drive towards a less adversarial construction industry is also clear in Australia. In 2018, the New South Wales Government released its ‘NSW Government Action Plan- a ten-point commitment to the construction sector’ (New South Wales Government, 2018). In 2020, notions of good faith and collaboration were brought to the fore by the release of empirical studies and reports on the construction industry and its current adversarial state (Australian Constructors Association, 2020; Sharkey et al. 2020). The Australian Constructors Association is currently actively promoting the use of more collaborative commercial frameworks but these need ‘to align the interests of all parties to the greatest extent possible and be drafted to achieve best for project outcomes rather than favouring any one particular stakeholder’ (Australian Constructors Association, 2020a: 9). The Association is also recommending a ‘playbook’ for the industry. This trend is gaining momentum in 2021 with the release in Australia of the NEC 4 suite of contracts, and the launch by the federal government, of the 2021 infrastructure plan points to the need to enhance project outcomes by reducing risk and improving value for money by using common and best commercial arrangements, standard form contracts and a delivery approach to infrastructure (The Australian Government, 2021: 269). State and territories are presented as proposed leaders in implementing this recommendation. Beyond the construction industry, industry codes of conduct are appearing almost yearly and regulate particular long-term contracts with an explicit duty for parties to act in good faith.^16^

This trend for a more collaborative industry is developing at a faster pace than the common law. The legal framework to support the less adversarial and more collaborative environment sought could helpfully be underpinned by the ‘relational’ contract theory. However, after thirty years of discussion (and 9 years since *Yam Seng*), it appears to be as difficult to embed these concepts in construction law practice as it is in the wider law. This *party centric* approach exacerbates the cycle of rejection noted above and creates a doctrinal gap. This gap in turn weakens any chance of building a proper legal framework. The result of all this leads to more detailed and complex written agreements. These are firstly, to specify and identify all that might be needed to deliver the project to define the baseline for further work in clear - and indeed exacting - terms, and secondly, to set out and agree to steps that might govern changing circumstances and conflicts. Although this forces parties to plan ahead and think through matters, there is increasing scope for errors or differences in interpretation to emerge, in turn putting an even higher burden on contract managers and opening up the scope for disputes. Simply put, seeking to define and specify further meaning to contracts to capture the same sort of ideas as “good faith” might provide more words to interpret and argue about.

Thus, the failure to develop the concepts of relational contract and good faith in case law – and the circle of rejection it causes - not only fails to recognise the growing idea of contracts in construction policy and practice but also that the contract is not solely an adversarial process (Gounari 2021: 182), but also ‘a cooperative endeavour’ (Finn 1989: 76). This is serious since it ‘hinders the reality of the relation’ (Gounari 2021: 182), which therefore creates a ‘fissure between the law on the book and the law on the ground’ (Gerhart 2020: 95). To fill this ‘fissure’, it is crucial to move the debate beyond whether or not good faith has a place in contract law, to how to recognise and utilise it. To do so, we propose the replacement of the existing *party-centric* approach by one which is *project-centric*. Consideration of the literature and the case law demonstrates that this would involve an evolutionary development rather than a revolutionary one and would help move the law closer to the more collaborative policy goal.

## Defining the project-centric approach

The *project-centric* approach is yet to be fully recognised or articulated by judges. Yet, it can be seen in, amongst others, *Sheikh Al Nehayan v Kent*,^17^*Amey Birmingham Highways Ltd v Birmingham City Council*^18^ and *Bates v Post Office Ltd*^19^ where the courts recognised the parties’ common purpose in seeing the contract performed.

In essence, the *project-centric* approach revolves around the following founding statements:

1. It is an approach which is relational in its basis and requires the parties to act in good faith. This requirement can emerge from an implied term of good faith – or the use of express obligations of good faith.
2. The approach relies on bringing together the internal “legal” content of good faith with its wider context.
3. Consequently, the existing, understood content of the rights and obligations flowing from good faith are required.
4. The further issue of filling the ‘fissure’ is helped by ensuring that the conduct of the parties within the wider relational framework is interpreted in a *project-centric* way.
5. Flowing from this, the contract itself is to be interpreted in a *project-centric* way. This is linked to – but different from – a purposive interpretation.

In short, we consider the dictum of Lord Justice Jackson in *Amey v Birmingham* articulates how relational contracts should be treated and that dictum should form the foundation of the way forward. In that case, he said:

Any relational contract of this character is likely to be of massive length, containing many infelicities and oddities. Both parties should adopt a reasonable approach in accordance with what is obviously the long-term purpose of the contract. They should not be latching onto the infelicities and oddities, in order to disrupt the project and maximise their own gain.^20^

It echoes Jackson LJ’s earlier extra-judicial suggestion that there should be a ‘bold’ approach where there is an ‘express’ obligation of good faith in a contract: “*to be slightly more willing to give effect to the obvious purpose underlying the contract”* (Jackson 2017: [6.10]). Without wishing to give a strained interpretation to the wording in a lecture, the “slightly” is telling here. It suggests that only a relatively minimal change in approach is required. We agree. Taking that step would draw upon existing authority and the current direction of travel in policy terms. Yet, despite this – and despite the relatively limited additional requirement on the parties – the step is not being taken. We therefore argue that although this step might appear small, it would nevertheless be significant in its impact.

The *project-centric* approach puts the delivery of the parties’ agreed outcome, i.e. the project, at the centre of the operation of the contract. The agreed objective is the reason for the parties’ contractual relation. It is therefore not independent of the contract but very much part of the contract and highly relevant to understand the parties’ duties to each other and to the project: it is what (contractually) brings the parties together. The *project-centric* approach helps to articulate the shared values that the parties have in the project, beyond their own respective contractual obligations. It is therefore crucial to understand how relational contracts and good faith, as concepts, might be synthesised from the academic approach to the existing policy and practice.

The next steps are then to explain (A) how the *project-centric* approach, as outlined above, operates within the context of the discussion of broader contract theory, and (B) how it would apply within the developing understanding of relational contracts in construction law. Thirdly (C), the interaction between the *project-centric* approach and the existing case law is discussed.

### Applying the *project-centric* approach within contract theory

Good faith and relational contract, as concepts, transcend existing contract law theories as we know them. Relational contract is a concept where contract law and wider cultural considerations interact. Good faith, as a concept, is infused with ‘other-regarding values’ (Gerhart 2020: 95) which are not clearly articulated in traditionally *party-centric* values of contract law theory. The courts are sensitive to context and the reasonable expectation of the parties when interpreting contracts, but this is still not enough. The work of Mitchell is relevant here – in particular her placing of the formal, legal contract within its wider relational context is important. We agree that ‘to truly embrace the relational approach, interpretation can not only consider the contractual but the entire relationship’ (Mitchell 2013: 239). This means not just interpreting the agreement but also finding a way to account for externally-generated notions (that is, those which arise from outside of the parties’ written agreement) such as good faith. By only recognising ‘internally generated norms’ and not ‘externally generated’ ones (Mitchell 2013: 239), the courts are closed to the possibility that ‘the wider context and the norms generated by it are relevant to how the contractual relation should be understood, obligations derived and interpreted, and disputes resolved’ (Mitchell 2013: 238).

The discussion on ‘externally generated norms’ clashes with the idea that courts should not use their own judgement to rewrite a ‘bad bargain’, and so is met with resistance in both the UK and Australia (although the manner in which this is dealt with in the different jurisdictions is slightly different.) In the UK, the courts tackle this challenge through a binary distinction to contractual interpretation: as either textual (within the written agreement) or contextual (looking more widely). Mitchell argues that to say that there is a ‘real deal’ and a ‘paper deal’ perpetuates a binary divide, which is false (Mitchell 2013: 240). The problem faced by the judiciary lies in the inherently adversarial norms of *party-centric* contracting that prevent the extent of these distinctions being understood and then engaged with. Indeed, given the role of the law in respecting the ‘parties’ expectations’, the link must be acknowledged. We ought to think of ‘contract and relations as related by distinct institutional frameworks’ (Mitchell 2013: 98). Arguably, the law in its *party-centric* approach only acknowledges the contract and the legal obligations that derive from it as a source of norms. This only sees the parties’ individual obligations, based on their own self-interest. In Australia, there are questions still be to answered as to the place of the intention of the parties in the interpretation of written terms. The court will have to ascertain what the parties have intended. In application of the parol evidence rule, once parties have agreed to the contract in writing, extrinsic evidence, the so-called factual matrix is excluded, unless there is ambiguity.^21^ Whether this is still applicable in commercial contracts is yet to be resolved.^22^

Both approaches are too restrictive. As a contract is part of and within the wider framework of the ‘relation’, that wider framework needs to be acknowledged as a source of norms, too. This is where the *project-centric* approach helps to decouple the parties’ individual obligations (as contractually defined) from the wider purpose of the relation (Robertson 2019: 234), and, we argue, the project. It is indeed ‘artificial to separate the legal obligations from the relational context’ (Corcoran 2012: 12). However, that relational set of norms also needs its own tools. The NEC contracts, codes of conduct and other policy and practice driven initiatives with the *project-centric* approach help towards this wider cultural view. They link the law with its wider socio-cultural-economic context. These aim to guide the parties’ performance of their obligations and not just define them. Thus, the *project-centric* approach provides a wider, more flexible context for following the instructions contained within the contract.

However, that wider context nevertheless remains focused on the parties’ agreement.The project itself is something that they have agreed upon. Thus, the norm is immune from the critique of reference to “commercial common sense” as a tool for interpretation of contracts.^23^ It relies on the parties’ agreed intention rather than something superimposed from outside. That outside context is nevertheless crucial as a tool for determining and interpreting any other obligations flowing from and to it; in particular, the good faith obligations of the parties. The parties’ individual obligations are seen within the wider purpose of the relationship. Good faith is owed not to each other but the agreed purpose (Collins 2016: 51). In short, repeating the mantra that good faith is the standard of cooperation to achieve the agreed-upon result (Corcoran 2012: 9).

In providing a context for following the instructions contained within the contract, the *project-centric* approach straddles both law and practice. One reason why the notion has resonance within construction law is because that area has been traditionally one where practice has been given particular weight. As Lord Dyson says, it was traditionally considered that construction law was ‘all about the facts, not about the law’ (Dyson 2016: 160). The *project-centric* approach, which requires consideration of both the terms of the contract and the actions which are required by those terms, fits well within construction law.

However, there is a danger that attempts to explain the approach without articulating the underlying concept fall into the fissure between doctrine and practice described above. Instead, ideas pile up on either side of the fissure, making it deeper rather than filling it in. Relational contract, as a concept, provides the bridge. Relational contract is therefore the foundation to give effect to the framework of *project-centric* approach and move away from the either/or distinctions of the current approach. In short, the *project-centric* approach allows for the recognition of a ‘relationally constituted contract law’ (Mitchell 2013: 243)) which recognises legal and wider norms as binding the parties.

Relationalism, linked to good faith, is the foundation which gives effect to the *project-centric* approach recognising that, in certain circumstances, the parties’ competing interests are nevertheless merged in the adoption of a common set of goals. ‘Once a particular aim is recognised as a contractual purpose then it can inform the interpretation of the contract’ (Robertson 2019: 235). This is where the link between relationalism and good faith is most relevant. Good faith as a concept is not autonomous since ‘its application depends on two issues relating to the context, the characterisation of the relation or activity where good faith is inserted and the nature of the legal obligations in which good faith obligations must be interpreted’ (Corcoran 2012: 100).

This role, and the interaction between relational contract and good faith are exemplified by Professor Collins’ summary of the main components of such a contract as follows:

1. A long term business relationship that will **provide sufficient pay-offs to both parties** to continue with the relationship even through periods of considerable adversity.

2. **Obtaining the benefits of the business relationship** will require adaptation, cooperation, and evolution of performance obligations, so that indeterminate implicit obligations of this kind **must be central to the deal.**

3. These implicit indeterminate obligations must be understood as arising not from general moral standards or norms of reciprocity such as honesty, **but will be tailored to achieve what is necessary to secure the success of the venture**. Business necessity in this context requires acceptance of obligations derived from the general concepts of cooperation and loyalty or commitment to the project (Collins, 2016: 43). (emphasis added)”

This makes the project vital to the relationship – but makes it clear that this also benefits the parties individually and that it helps to measure the obligations of good faith owed to it. It helps to reconcile the individual interest of the parties with the wider purpose of the contract. As such, it recognises the reality of the contracting experience, that a rational party can be ‘pursuing their own self-interest in the contract’ whilst also ‘agreeing to cooperate as a way to achieve that objective’ (Gounari 2021: 183)).

The focus of the enquiry to determine whether the contract is relational is therefore to see what brings the parties together. For example, a (long-term) project that both parties are heavily invested in (for some, all contracts are therefore relational in some way (Eisenberg 2000:821)). The implicit obligations of cooperation and loyalty of commitment are owed to the project and not to each other. Depending on what the project is, those implicit obligations necessary to achieve the success of the venture will vary. The context is therefore important. This *project-centric* approach can also be gleaned from *Yam Seng*, *Bristol Ground School v Intelligent Data Capture Ltd*^24^ and *D&G Cars Ltd v Essex Police Authority*^25^ (what Collins referred to as ‘the trilogy of relational contracts’ (Collins 2016: 39)). In all these cases, the breach was established when one party failed to act for the success of the project and instead acted for their own interest. This shows how good faith, as an ‘other-regarding value’ is relevant to assess the duties of the parties by taking a *project-centric* approach. This is important and these dimensions were refined by Leggatt J, in *Sheikh Al Nehayan*, when he said that what ‘was intended to be a long-term collaboration’ in which the interests of the parties ‘were inter-linked’^26^ was a ‘classic example of a relational contract’.^27^ This link between the parties’ interests, we argue, highlights the *project-centric* approach. Leggatt J continued, ‘while the parties to the joint venture were generally free to pursue their own interests and did not owe an obligation of loyalty to the other,’^28^ anything that prevented this common purpose would be a breach of good faith.^29^

Our proposal to give effect to a *project-centric* approach is born from the promise of the parties to the bargain, its performance and the benefits the end project will give to the parties. This is not entirely a novel approach in itself since it appears to be implicitly guiding the reasoning of the courts in construction contracts and also exists, to some extent, in the purposive approach (Robertson 2019: 230). Yet implicit guidance is not enough. We now turn to the construction examples to show how to distil, explicitly, what the courts do into a working tool.

### Applying a *project-centric* approach to construction law

The emerging nature of collaborative construction contracts places them particularly closely to the crucible in which ideas of relational contracts and good faith are developing. The various policy and practice innovations are sketched out above, reflecting continuing movement towards increased commercial flexibility and managing conflict within contracts. That said, examples of the sort of contractual frameworks identified have been said, themselves, to demonstrate the relational quality of construction contracts (McInnis, 2003; Circo 2014). There is, however, a question surrounding whether construction contracts would fit the criteria for a relational contract established in *Bates*. As Fraser J noted, these criteria are not exhaustive and not all relational contracts would comply with them, except perhaps the very first criterion that the agreement must not contain specific express terms in the contract that prevent a duty of good faith being implied into the contract.^30^ So, the question remains an open one for construction contracts.

Shy Jackson, who has written extensively on good faith (Jackson 2017, 2018, 2019) has provided a useful summary of this from a practitioners’ perspective in a client focused briefing on the *Bates* criteria:

Indeed, the increasing use of collaborative models is driven by the recognition that construction projects, by their nature, require close cooperation over a lengthy time period in order to deal with the inevitable risks that arise on such projects. If a contract to distribute Manchester United-branded toiletries in the Far East was considered a relational contract, as in the *Yam Seng* decision, it is difficult to see why a construction contract - especially one in which the parties chose to use a collaborative form of contract - will not be seen as a relational contract. (Jackson, 2019).

It is obvious that some *Bates* criteria will apply to some construction contracts. It is not certain that they will in every instance. As Jackson notes above, construction projects are long-term contracts and can involve the management of significant levels of change – two of the key hallmarks of a relational contract. However, there are also distinctions. For instance, while the construction contract can be performed over several years, this is aimed at a particular end point: the delivery of the ‘thing’ contracted for. In this sense it is closer to a classical contract than a relational one. It distinguishes the type of contract agreed for the building of a factory, which will make Manchester United-branded products from the contract for the distribution of those goods. In the former, the contract will end successfully when the project is delivered. In the latter, there is no end point: a successful relationship could continue indefinitely. (It is also, of course, distinct from the eventual contract formed for the purchase of those goods by consumers.) The construction contract will usually bear some aspects of the relational contract.

Acknowledging that relational contracting is a spectrum (Macneil 1974: 736-7), construction contracting models can be found at all points in the spectrum. At one end, we find so-called modular construction. In this situation, the bulk of the construction work is carried out by the contractor away from the eventual location of it. This generates a kit or even pre-constructed product, which is then delivered to site and installed. While that transaction perhaps takes longer to complete than a simple sale of goods, it has the elements of a classical discrete agreement, based on the provision of a product by a seller to a buyer. At the other end of the spectrum there are joint venture agreements which embody the principles of a partnership and have parties’ commercial interests aligned. These parties have fiduciary duties to each other and may go beyond the sort of relational contract envisaged in *Bates*.

The third category that sits across the middle reaches of the spectrum is the most difficult to categorise as these contracts can rarely be described with one particular label. Some may meet the *Bates* criteria and some may not. Increasingly, however (in response to the commercial and policy drivers noted above) these contracts include some form of ‘relational’ provisions within them. That might amount to express good faith obligations, detailed communications mechanisms, pain/gain share provisions, early warning mechanisms, early contractor involvement, enterprise agreements and the sort of complex enumeration of rights and obligations and which incentivise and support such mechanisms as the parties might agree within the scope of their own agreement, which terms are of course offered without limitation. This then places construction contracts in a position where they have a number of ‘relational’ features. Many of these features are expressly provided for within the contract. We argue that these features facilitate a *project-centric* approach by aiding communication and providing mechanisms to address changes within the project. These are helpful on their own but do – of course – add complexity to the contract administration. The *project-centric* approach would apply where the parties’ contract was interpreted as having sufficient relationality – whether expressly agreed or implied from the wider context of the agreement to merit it.

It is therefore so important for that reason that there is some form of broader obligation, which can help facilitate these mechanisms. That broader obligation aligns with values such as good faith, cooperation and other relational values. The crucial importance of values such as good faith is perhaps best exemplified by the fact that where there is doubt about whether the contracts meet the *Bates* criteria for a relational contract, many construction contracts attempt to resolve such doubt by providing for express good faith obligations. As with the broader debate on relational contracts and good faith, the difficulty of then articulating the content of the good faith obligation poses a problem.

One of the key points made against implied terms of good faith was by Sir Rupert Jackson saying that, “[parties] all need to know what the contract requires and what the contract permits. To that end, they do not speculate about ethics or metaphysics. …. They look at the black letter provisions of the contract. That is what the court should do as well” (Jackson 2017: [6.11]).

This is true and militates against the implication of ‘good faith’ in a contract. More generally this criticism might be levelled against using the concept of good faith itself. However, it does not address what should be done when the contract itself contains blackletter provisions creating obligations of good faith. What is then needed is a practical and understandable approach to interpreting this good faith provision.

There are two steps to this. Firstly, the relational character of the construction contracts should be given meaning and effect – especially where there is an express good faith obligation in the contract representing the parties’ agreement to incorporate aspects of relationalism. That meets the parties’ intention. The existing values which arise from good faith, such as honesty, fair dealing and not acting capriciously^31^ should therefore be recognised and enforceable. Embedding these values will assist in managing the complex provisions for dealing with change in construction contracts. Thus, the implied obligations which arise from relational contracts should be part of – or at least aligned with - the *project-centric* approach.

The second point arises because the current definition still leaves the ‘fissure’ between law and practice identified above. It poses the broader issues of parties understanding what it is that the contract needs them to do. One of the key ways to ensure that there are fewer disputes is to make sure that the contract is well understood by those who use it. We argue this goal is not necessarily assisted through increased complexity and volume of the documents attempting to create processes to deal with different issues and/or defining terms ever further. Rather simplification and clarity should be the aims. That is helped by an approach which intuits something about how things should be understood. Sir Rupert Jackson – and others – have correctly identified that the resort to the blackletter terms of the contract should not go as far as relying on technicalities.^32^ However, we argue that where contracts contain an overarching duty of good faith (whether express or implied), the judicial reticence to give effect to it seems to cut against the need to give effect to the parties’ bargain. Courts have the ability to resolve such disputes, even in the context of the ‘adversariality’ often present in construction contracts, through a *project-centric* approach where the ‘other regarding’ context – anchored on achieving the parties agreed project – is taken into consideration.

These developments have happened within the more sceptical discussion of good faith. Within the construction context specifically, Lord Justice Coulson and Sir Rupert (formerly Lord Justice) Jackson, both senior construction judges, have both voiced somewhat sceptical views of good faith and relational contracts as concepts (even while Sir Rupert gave the best articulation of a *project-centric* approach in *Amey Birmingham.*^33^) This can be seen in some of Sir Rupert’s remarks, noted above, and the comment by Coulson LJ of good faith being something of only academic interest (Coulson 2019). However, in both cases the substance of the concern lies within the choice of words rather than the underlying policy. *Indeed*, it is striking that both Jackson (Jackson 2020: 7) and Coulson (Coulson 2019: 9) view cooperation as fulfilling part of the need. This still leaves the question of how far cooperation goes. Indeed, cooperation is, in itself, a relational value and somewhat open textured in terms of how it might be understood.

To the extent that the answer to how good faith is used can be found within the terms of the contract itself: we agree. As the following case analysis shows, parties have to cooperate to ensure the project is delivered. Using a *project-centric* approach helps in determining the boundaries of good faith and relational contract by using other-regarding values. It is worth repeating, good faith (understood as a standard of cooperation (among other things)) helps in achieving the agreed upon objectives (Corcoran 2012: 9).

### Applying the *project-centric* approach within the existing case law

The *project-centric* approach and its attempt to give substance to the idea of the relational contract and good faith may seem on its face to be somewhat esoteric but its centrality is clear when the judicial and academic discussion of the definition of good faith are considered. The need to focus on the aim of the contract comes across clearly in the leading cases, to which we now turn. An analysis of case law shows that there is already the articulation of an attempt to break the vicious circle of rejection we have laid out above, and that this uses the language of the *project-centric* approach. The key is to recognise and emphasise this. As noted above, this can be seen in cases such as *Amey v Birmingham City Council*^34^ and *Sheikh Al Nehayan v Kent*^35^ but also seen in *Bates v Post Office Ltd (No3)*,^36^ by explicitly recognising the parties’ common aim in seeing the contract performed. The following paragraphs present judicial decisions that are advancing a *project-centric* approach, albeit not consciously. An analysis of the Australian and English case law highlights the dialogue between the two jurisdictions. This is particularly true in the few relevant construction cases in the UK. While the following discussion presents cases of each system of law, we have also decided to highlight the judicial conversation between Australian and English judges which is present in both contract (generally) and construction cases.

This *project-centric* approach started to appear in the New South Wales Court of Appeal decision of *Renard Construction v Minister for Public Works* (1992).^37^ Renard Constructions was contracted to build pumping stations for a sewerage project in New South Wales. By focusing on the infidelities of the contract, the principal did not adopt a *project-centric* approach, and this came to the fore in litigation.^38^ This was an instance of a discretionary right not exercised reasonably. Priestley JA also reflected, in obiter, on reasonableness and good faith, and highlighted the resemblance between the concepts, describing them as ‘standards of fairness, and community expectations’.^39^ Priestley JA’s obiter would become most commented upon (Peden 2003; Carter et al., 2003; Warren 2010; Dixon 2011). Some subsequent cases used the obiter to recognise an implied term of good faith, such a term consequently limiting the exercise of a discretional right. This *project-centric* approach has also been adopted in *Bundanoon v Cenric*^40^ where once again, the principal had already made up its mind by the time the notice was issued, thereby breaching an implied duty to act in good faith.^41^ Later decisions have used *Renard* to imply a duty to act in good faith in the performance of a discretionary right in different contexts.^42^

The Federal Court of Australia has considered good faith numerous times,^43^ but has yet to enforce it.^44^ Australia is still awaiting a decision of the High Court on the status of good faith in Australian contract law. In 2015, the High Court of Australia heard a dispute on the validity of late payment fees for credit cards.^45^ Allsop CJ took an opportunity to summarise good faith as:

1. an obligation to act honestly and with fidelity to the bargain;
2. an obligation not to act dishonestly and not to act to undermine the bargain entered or the substance of the contractual benefit bargained for; and.
3. an obligation to act reasonably and with fair dealing having regard to the interests of the parties (which will, inevitably, at times conflict) and to the provisions, aims and purposes of the contract, objectively ascertained.^46^

In each of these three points, the idea of the underlying centrality of the bargain, or the agreement, emerges strongly. This summary is taken from the definition of good faith provided by Sir Anthony Mason (Mason 2000: 66). Four years later in the UK, in *Sheikh Al Nehayan v Kent*, Leggatt J determined that

…this summary is also consistent with the English case law as it has so far developed, with the *caveat* that the obligation of fair dealing is not a demanding one and does no more than require a party to refrain from conduct which in the relevant context would be regarded as commercially unacceptable by reasonable and honest people.^47^.

Most recently, Fraser J endorsed Legatt J’s approach in *Bates*.^48^ The judicial discussion between the UK and Australia in mainstream contract law is therefore clear and is also present in construction cases to which we turn.

A *project-centric* approach recognises the need for a flexible attitude to successful contract performance, reliant on the contextual backdrop of the agreement. Indeed, shared expectations go beyond the contract terms (Gerhart 2020: 98). In Australia, this idea was highlighted in *Automasters Australia Pty Ltd v Bruness Pty Ltd*, decided by the Western Australian Supreme Court,^49^ where the retention of a report and decision to issue a notice for default without careful analysis demonstrated a lack of good faith, the franchisor having made up their mind to terminate the agreement.^50^ In addition, good faith was said to:

import a duty to have due regard to the legitimate interests of both parties in the enjoyment of the fruits of the contract. In some circumstances a cynical resort to the black letter or literal meaning of a contractual provision may be taken into account in determining whether there has been a lack of good faith.^51^

The importance of context and the flexible nature of good faith was also highlighted. ‘What constitutes good faith will depend on the circumstances of the case and upon the context of the whole of the contract.’^52^ The key step is then developing what this means in terms of practice. It should not simply lead to further debates on definitions but provide a tool to be considered by the judiciary.

The same year the New South Wales Supreme court rendered its judgment in *Overlook v Foxtel*.^53^ Barrett J considered how selfish a party can be before potentially breaching a standard of conduct to act in good faith.^54^ Considering Peden’s work, Barrett J stated that:

the implied obligation of good faith underwrites the spirit of the contract and supports the integrity of its character. A party is precluded from cynical resort to the black letter. It is, rather, a duty to recognise and to have due regard to the legitimate interests of both the parties in the enjoyment of the fruits of the contract as delineated by its terms.^55^

This reasoning is not foreign to construction cases – especially when it chimes with a clear policy aim. Courts already take a robust view of parties’ conduct when it comes to the operation of payment provisions in contracts, and their enforcement through construction adjudication. So, for example, an ‘over-literal’ reading of the payment provisions in the UK security of payment legislation gave way to their clear purposive interpretation.^56^

The judicial dialogue between these jurisdictions can be seen from the specific discussion of the *Automasters* and *Overlook* Australian judgments in the English case of *Costain v Tarmac Holdings Ltd.*^57^ In this case, then Mr Justice Coulson was asked to consider the scope of the “mutual trust and cooperation” clause within the NEC 3 standard form of contract in considering a dispute about the extent to which one party to a contract might be obliged to correct the other party’s apparent misinterpretation of the dispute resolution provisions. While he recognised that there is some content to be given to a good faith obligation, he said that it ‘did not require the parties’ to act against their own self-interest.^58^ In saying this, he echoed the understanding of good faith as set out in the textbook *Keating on NEC 3*. (Thomas, 2012) In summary, these reasons delineated good faith narrowly but set out, as a conclusion in this exercise, that the duty is one ‘to have regard to the legitimate interests of both the parties in the enjoyment of the fruits of the contract as delineated by its terms’.^59^ In *Costain*, Coulson J commented on this saying he was broadly in agreement ‘although … a little uneasy about a more general obligation to act “fairly”; that is a difficult obligation to police because it is so subjective.’^60^

We argue that a *project-centric* approach brings the objectivity needed to help determine whether a party has acted in good faith – and therefore met the requirements of the contract. We also argue that whether the parties’ enjoyment of the fruits of the contract is overtly affected can be considered objectively in light of the facts of the case and the context of the transaction.

From the above, there is a clear strand within the Australian and UK case law which can be seen to be speaking to a *project-centric* approach: taking a view of giving effect to the parties’ bargain, and more generally supportive of a broader view of the contractual approach. However, it has yet to be fully understood how ‘the fissure’ between the law and the practice can be bridged. The work of Tan is helpful in this matter. It sets out the framework and criteria that might apply to the different ways in which doctrinal development of good faith could occur. Let us therefore turn to it.

## Putting the *project-centric* approach into practice – issues of theory

### The doctrinal basis of a *project-centric* approach

Tan (2019) has discussed the emerging discussion on relational contracts and has identified three ways in which it could develop. These are re-interpretive relationalism (Tan, 2009: 105–107) where existing rules and doctrines are reinterpreted along relational lines; re-orientative relationalism, (Tan, 2009: 107–111) described as the ‘process of making explicit salience and additive changes to the content, structure and priority of rules and standards within a doctrine’ (Tan, 2009: 105); and reconstructive relationalism, (Tan, 2009: 111–116) described as a more complete overhaul of contract law. The analysis of the *project-centric* approach for relational contracts, or those incorporating express obligations of good faith, would fit within the second category. Re-constructive relationalism runs in line with the policy push within the construction industry – to remake the way in which the culture runs. However, this meets the more conservative approach in the case law, which looks to develop the law without legislative change, and without the necessary development of language. Therefore, there is some tension between these two drivers for change and the result is the complexity of the options discussed here.

The re-orientative approach provides the best explanation of the way to develop the *project-centric* approach as a response to judicial and construction industry developments. The reorientation also facilitates the bringing in of ‘standards’, through good faith, which can give rise to implied terms – in particular good faith - and which have a ‘higher normative demand’ (Tan 2019: 110). Increasing the salience and weight placed on the requirement for the parties to meet the agreed common purpose, and developing that by giving a standard to which the parties should be held would act as a means of developing the existing understanding. It would also fit the existing, articulated prism of meeting that agreed common purpose. On the analysis put forward, this gives effect to the under-emphasised characteristic of relational contracts (and indeed potentially all contracts) while using existing concepts to meet the recognised normative standards which are clear from the construction industry’s contracting developments.

From the above, the context of the construction industry efforts to map out the cooperative aspect of contracting is crucial. The industry is creating a new vocabulary which builds on the understanding and definitions which the courts are trying to articulate but cannot because they adopt a *party-centric* lexicon, as opposed to a *project-centric* one. This is not only important in relation to implied obligations but is also linked to contract interpretation. The debate surrounding the ‘relational contract’ has always been linked to the contextual enquiry (Mitchell 2013: 238).

Our framework, when put into practice, shows that it is linked, but nevertheless different from the purposive interpretation (Robertson 2019).

### Moving towards a project-centric interpretation of contracts and bridging law and practice

The Supreme Court in *Wood v Capita Insurance Services Ltd*^61^ stating that the interpretation was an ‘iterative process’^62^ seems to indicate that the courts are ready to move towards a *project-centric* approach. This iterative process is important to highlight ‘the fact that contractual purposes are bilateral does not mean they are conflicting … the contract as a whole, and any individual provision, may be understood to represent an accommodation and reconciliation of two competing sets of interests’ (Robertson 2019: 234).

Interestingly, the purposive approach was recently explicitly recognised in the Scottish case of *Ardmair Bay Holdings Ltd v James Douglas Craig*.^63^ The Inner House, treated a purposive interpretation to the contract as self-evident and considered external norms (in this case commercial knowledge) to be relevant in interpreting contracts. Moreover, there is a consistency of approach – the purposive interpretation clearly echoes the approach suggested by Jackson LJ in *Amey v Birmingham*, to keep focussed on the ‘fundamental purpose’ and not be distracted by infelicities in drafting.^64^ We agree, but suggest that *project-centric* good faith, rather than purposive interpretation, is a better both norm to consider as it is anchored in the parties’ agreement and links it to external norms.

The *project-centric* approach helps to do so by focusing on what unites the parties (project) rather than what separates them (individual interests). Although the interpretative process does hint at this through the purposive approach (Robertson 2019), it is not yet firmly established as a working tool which therefore prevents its wider application. We argue that this approach can be a conduit to give effect to the wider context that needs to be taken into consideration. In *Commonwealth Bank of Australia v Barker*,^65^ Kiefel J’s obiter on good faith is only an indication that good faith could be considered as a standard of conduct rather than a fixed rule.^66^

Although purposive interpretation allows the separation of the individual interests from the core purpose of the contract (Robertson 2019: 234) and highlights the *project-centric* approach, it however does not go far enough in inserting ‘other regarding values’ such as good faith or recognise the relationality of the contract. By formally recognising the *project-centric* approach, courts and contractual parties are given the vocabulary to recognise all norms and values which form part of the contractual journey. Moreover, while the external values are inserted into the discussion, they remain rooted in the parties’ agreement. The project is defined by them. Beyond that, in many projects, because of the multiparty network of contracts there is a necessity for the project to be defined outside of the individual contract (there will be a design for the construction of the eventual house, factory, hotel etc. which will be the basis for the project). If there is a large and complex public-private partnership for the construction of a hospital then the sub-sub-contract for electrical installation will be being carried out by reference to the same project (even if only part of it) as the agreement between the financial institutions which form the funding special purpose vehicle. This is not to be overly technical on the nuance and definition of project in the formal sense – each contract will be governed by its own terms. Moreover, it is not to suggest that there is some anthropomorphic entity ‘the project’, which develops its own rights. That would be to move the focus of the discussion away from the parties and onto something else. Rather, the *project-centric* approach interpretation focusses on an interpretation of the parties’ agreement, which benefits the project – assessed in broad terms.

Raising the salience of the *project-centric* approach also highlights the crucial role of the approach as a tool to improving the articulation between law and practice on three levels. First, it gives effect to the relational element of the contractual journey and breaks the binary distinction in contract law between internal and external elements. As such it relieves the artificial tension between ‘individual interests and the agreed purpose’ (Robertson 2019: 234). Both are important but in different ways, they are therefore not in conflict.

Second, the *project-centric* approach is capable of intuitive understanding and pithy expression. This both helps resolve issues at the outset and during performance when problems occur. It shows that good faith and relational contracts, although complex ideas, can be applied and given effect to by the *project-centric* approach. Finally, it sits at the crux of law and practice and to some extent defies categorisation. However, given the undeniable link between interpretation and implied terms (Robertson 2019: 230; Robertson, 2016), it equally applies to both.

Thus, the *project-centric* approach brings together the various threads, which have highlighted for a while the limits of a purely adversarial position as not showing the whole contractual experience and the artificiality of ‘separating the legal obligations from the relational context’ (Corcoran 2012: 12). The *project-centric* approach gives effect to what Mitchell was articulated: that it is not only that contract law must follow commercial law practice but that these practices must fit within a legal framework (Mitchell 2013: 441).

## Conclusions

The debate in the construction industry in both the UK and Australia demonstrates that a *party-centric* approach to contracts is not necessarily applicable to all commercial dealings. The development of collaborative frameworks, new industries and policies have highlighted the inadequacy of holding to a purely traditional perspective of contract law of parties as adversaries, and that instead a *project-centric* approach, based upon good faith and relational contract, would better reflect the reality of the contracting experience as a more cooperative experience (Gounari 2021: 182). Although present in some cases, the centrality of the project is understood but not articulated. The current hesitation of the courts relates to the place of these doctrines but also the lack of vocabulary and framework surrounding the two notions of good faith and relational contract.

We argue that the *project-centric* approach is a means by which to provide a fresh approach to the ideas of good faith and relational contract. We are therefore proposing to re-orientate the debate and finally acknowledge the doctrinal impact of both doctrines as a basis upon which we can bridge law and practice. Crucially, it meets a practical, policy need within the construction industry in both the UK and Australia.

## Footnotes

1 We are restricting our enquiry to England and Wales. For the Scottish position, see H MacQueen and S O’Byrne ‘The principle of good faith in contractual performance: a Scottish-Canadian comparison’ (2019) Edin LR 301–331.

2 The British Institute of International and Comparative Law, (2020) Concept Note 2 on the effect of the 2020. pandemic on commercial contracts, available at https://www.biicl.org/breathing-space [accessed 02 September 2020].

3 [2013] EWHC 111 (QB), [2013] 1 All ER (Comm) 1321.

4 [2018] EWHC 333 (Comm).

5 [2019] EWHC (606) QB.

6 The two notions of good faith and ‘relational contracts’ appear to have a ‘marmite’ effect on the English courts with some embracing them wholeheartedly and others resisting them in no uncertain terms. In favour of relational contracts, *Amey Birmingham Highways Ltd v Birmingham City Council* [2018] EWCA Civ 264, *Bates v PO Ltd (No3)* [2019] EWHC (606) QB, *Essex CC v UBB Waste (Essex) Ltd* [2020] EWHC 1581, [112] (TCC) where it was held that the contract under consideration was ‘a paradigm example of a relational contract in which the law implies a duty of good faith’. Rejecting the notion: *UTB LLC v Sheffield United & Others* [2019] EWHC 914, *Russell v Cartwright* [2020] EWHC 41 (Ch), *Morley v RBS plc* [2020] EWHC 88 Ch for an ordinary loan facility agreement.

7 (1992) 26 NSWLR 234.

8 *Compass Group (UK and Ireland) Ltd (t/a Medirest) v Mid Essex Hospital Services* [2013] EWCA Civ 200, [2013] BLR 265; *MSC Mediterranean Shipping Co v Cottonex Anstalt* [2016] EWCA Civ 789, [2017] 1 All ER (Comm) 483 for good faith and *UTB LLC v Sheffield United & Others* [2019] EWHC 914, *Russell v Cartwright* [2020] EWHC 41 (Ch) for relational contracts in the UK, and cases such as *Royal Botanic Gardens and Domain Trust v South Sydney City Council* [2002] 240 CLR 45; *Commonwealth Bank of Australia v Barker* [2014] 253 CLR 169 in Australia.

9 (1766) 3 Burr 1905.

10 *Royal Botanic Gardens and Domain Trust v. South Sydney City Council* [2002] HCA 5, 240 CLR 45, [40] ‘whilst the issues respecting the existence and scope of a “good faith” doctrine are important, this is an inappropriate occasion to consider them’; *Commonwealth Bank of Australia v Barker* [2014] HCA 32, 312 ALR 356 [42].

11 *Compass Group (UK and Ireland) Ltd (t/a Medirest) v Mid Essex Hospital Services* [2013] EWCA Civ 200, [2013] BLR 265, [105] (*per* Beatson LJ); *MSC Mediterrannean Shipping Co v Cottonex* Anstalt [2016] EWCA Civ 789, [2017] 1 All ER (Comm) 483 [45] (*per* Moore-Bick LJ).

12 *Journal of Commonwealth law* (2018) Special issue; for Canada, *Bhasin v Hrynew* (2014) SCC 71 [93]; *C.M. Callow Inc .v. Zollinger* (2020) SCC 45.

13 It is beyond the scope of this paper to critique what relational contract theory is about (and even whether it exists as such). A good summary by Gounari is that ‘it has developed as a response to the perceived failing of classical contract which looks to the parties’ express bargain as paramount’ (Gounari 2021: 179). For details of reference of MacNeil’s work, see too Gounari, esp pp 179–181 and Tan 2019.

14 This has recently been confirmed as synonymous with good faith by the Inner House of the Court of Session in the judgment in *Van Oord UK Ltd v Dragados UK Ltd* [2021] CSIH 50, 2021 SLT 317.The precise content of that obligation was not discussed in detail but was considered a matter for proof. Ibid at [23]. As such there is no particular development of the concept in this judgment, albeit it represents a further step towards general acceptance of good faith. For discussion see (Christie: 2022).

15 *Housing Grants, Construction and Regeneration Act 1996* (as amended) ss 104 to 113.

16 *Competition and Consumer (Industry Codes – Dairy) Regulations 2019* (Cth), s 11; *Competition and Consumer (Industry Codes – Horticulture) Regulations 2017* (Cth), ss 8–9; *Competition and Consumer (Industry Codes – Food and Grocery) Regulation 2015* (Cth), s 6B; *Competition and Consumer (Industry Codes – Franchising) Regulation 2014* (Cth), s 6.

17 [2018] EWHC 333 (Comm).

18 [2018] EWCA Civ 264.

19 [2019] EWHC (606) QB.

20 Ibid. at [93].

21 *Codelfa Construction Pty Ltd v State Rail Authority of NSW* (1982) 149 CLR 337, 352.

22 Different perspectives can be found in *Western Export Services Inc v Jireh International Pty* [2011] HCA 45 [2]: ‘it is essential to identify ambiguity in the language of the contract before the court may have regard to the surrounding circumstances and object of the transaction’; *Electricity Generation Corporation v Woodside Energy Ltd* [2014] HCA 7 [36–37]: interepreting a commercial contract ‘will require consideration of the language used by the parties, the surrounding circumstances known to them and the commercial purpose or objects to be secured by the contract’; *Mainteck Services v Stein Heurtey* SA [2014] NSWCA 184 [69] where the court emphasised that ‘[w]ords do not have a “natural” meaning that can be determined in isolation.’; *Mount Bruce Mining Pty Limited v Wright Prospecting Pty Limited* [2015] HCA 37 which shows that the High Court of Australia is still split on the issue.

23 *Wood v Capita Insurance Services Ltd* [2017] UKSC 23, [2017] AC 173, [28].

24 [2014] EWHC 2145.

25 [2015] EWHC 223.

26 [2018] EWHC 333 at [173].

27 Ibid. at [174].

28 Ibid. at [176].

29 Ibid.

30 [2019] EWHC (606) at [725 − 26].

31 Ibid. at [702 − 25] (*per* Bates J).

32 *Amey Birmingham* [2018] EWCA Civ 264 at [93].

33 See discussion *supra* on *Amey Birmingham* accompanying footnotes 19–20.

34 Ibid.

35 [2018] EWHC 333 (Comm) at [173]–[176].

36 [2019] EWHC (606) at [725], the criteria for whether or not a contract is relational include “3. The parties must intend that their respective roles be performed with integrity, and with fidelity to their bargain. 4. The parties will be committed to collaborating with one another in the performance of the contract. 5. The spirits and objectives of their venture may not be capable of being expressed exhaustively in a written contract.” These draw from case law which consistently indicate some form of project centricity in their text.

37 (1992) 26 NSWLR 234.

38 Ibid. at [259].

39 Ibid. at [268].

40 [2019] NSWCA 87.

41 Ibid. at [160].

42 *Alcatel Australia Ltd v Scarcella* (1998) 44 NSWLR 349, 363–369; *Burger King Corporation v Hungry Jack’s Pty Ltd* (2001) 69 NSWLR 558, 566–573 [145]–[186]; *United Group Rail Services Ltd v Rail Corporation New South Wales* (2009) 74 NSWLR 618, 634–635 [58]–[59]; *Alstom Ltd v Yokogawa Australia Pty Ltd (No 7)* [2012] SASC 49.

43 *Marmax Investments Pty Ltd v RPR Maintenance Pty Ltd* (2015) 237 FCR 534, 561–563 [142]– [150]; *Paciocco v Australia and New Zealand Banking Group Ltd* (2015) 236 FCR 199, 272–274 [287]– [292]; *Virk Pty Ltd (in liq) v YUM! Restaurants Australia Pty Ltd* [2017] [2017] FCAFC 190 [167]–[185].

44 Reference to Allsop CJ’s judgment’s part in *Paciocco v Australia and New Zealand Banking Group Limited* [2015] FCAFC 50 was referred to in *Sheikh Al Nehayan v Kent*. Paciocco was also referred to in approval in *CPC Group v Qatari Diar Real Estate* [2010] EWHC 1535 (Ch) by Vos J.

45 *Paciocco v Australia and New Zealand Banking Group Limited* [2015] FCAFC 50.

46 Ibid. at [288].

47 [2018] EWHC 333 at [175].

48 [2019] EWHC (606) at [706].

49 [2002] WASC 286.

50 Ibid. at [392].

51 Ibid. at [388].

52 Ibid. at [148].

53 (2002) NSWSC 17.

54 Ibid. at [65].

55 Ibid. at [67].

56 *Melville Dundas (In Receivership) v George Wimpey* UK Ltd [2007] UKHL 18, [2007] 1 WLR 1136.

57 [2017] EWHC 319 (TCC), [2017] 2 All ER (Comm) 645.

58 Ibid. at [124].

59 Ibid. at para. 120.

60 Ibid. at [123].

61 [2017] UKSC 23, [2017] AC 173.

62 Ibid. at [12].

63 [2020] CSIH 21.

64 [2018] EWCA Civ 264 at [93].

65 [2014] HCA 32.

66 Ibid. at [105] (referring to Lücke 1987).
